# Validation of three mental health scales among pregnant women in Qatar

**DOI:** 10.1186/s12978-019-0806-6

**Published:** 2019-10-16

**Authors:** Katherine A. Roof, Laurie James-Hawkins, Hanan F. Abdul Rahim, Kathryn M. Yount

**Affiliations:** 1 Department of Global Health, Rollins School of Public Health, Emory University, Atlanta, Georgia; 2 Department of Sociology, University of Essex, Wivenhoe Park, Colchester, UK; 3 Department of Public Health, College of Health Sciences, QU Health, Qatar University, Doha, Qatar; 4 Department of Sociology, Emory University, Atlanta, Georgia

**Keywords:** Pregnancy, Depression, Anxiety, Validation, Arabic

## Abstract

**Objectives:**

The objective of this study is to validate three mental health scales in a targeted sample of pregnant Arab women living in Qatar: the Kuwait University Anxiety Scale, the Perceived Stress Scale, and the Edinburgh Postnatal Depression Scale.

**Methods:**

Random split-half exploratory factor analysis and confirmatory factor analyses (*n =* 336; *n* = 331), conducted separately, were used to evaluate scale dimensionality, factor loadings, and factor structure of the KUAS, the PSS, and the EPDS.

**Results:**

Fit statistics for the three scales suggested adequate fit to the data and estimated factor loadings were positive, similar in magnitude, and were significant. The final CFA model for the KUAS supported a 19-item, two factor structure. CFA models also confirmed 8- and 10-item, single-factor structures for the PSS and EPDS, respectively.

**Conclusions:**

The validation of scales for these aspects of mental health in Arab pregnant women is critical to ensure appropriate screening, identification, and treatment to reduce the risk of sequelae in women and their children. Findings offer a useful comparison to mental-health scale validations in other Arab contexts.

## Plain English summary

Mental health during pregnancy is important for the long-term physical and mental well-being of mothers and their children. Screening for common mental disorders, including stress, anxiety, and depression requires valid measurement tools carefully tested for the populations in which they are used. Valid measurements of mental health during pregnancy can facilitate appropriate treatment and follow-up.

In this study, we tested the validity of three scales for measuring anxiety (Kuwait University Anxiety Scale), stress (Cohen’s Perceived Stress Scale), and depression (Edinburgh Postnatal Depression Scale) in a sample of Arab pregnant women in Qatar waiting to receive antenatal care. For statistical analysis, the sample was randomly split in half (336 women and 331 women). Exploratory factor analysis was conducted on one half of the sample and confirmatory factor analysis on the second half. These analyses were conducted to assess the dimensions of each scale and how questions were related to the underlying concepts they are supposed to measure.

Our data supported a 19-question version of the anxiety scale with two dimensions reflecting cognitive/affective anxiety and somatic anxiety. The data also supported an 8-question scale for measuring stress and a 10 -question depression scale. Each of those two scales reflected one dimension only.

Our findings suggest that the tested scales can be used adequately in the population of Arab pregnant women. Differences with results from other samples emphasize the importance of carefully testing instruments for use in varied populations. Valid tools are needed for appropriate screening and subsequent treatment of mental illnesses.

## Background

Globally, mental disorders affect 10% of pregnant women and 13% of postpartum women [1]. Women from lower-income countries are at even higher risk for mental disorders; 16% of pregnant women and 20% of postpartum women [1, 2]. Failing to identify psychopathology in the perinatal period can lead to malnourishment of the mother and baby, stunted physical and cognitive development of the child, and in extreme cases, infanticide, or suicide [3]. Though validated and reliable screening scales exist for anxiety, stress, and depression, these scales have not been fully explored across Arab contexts, with diverse samples, including pregnant women.

The 20-item Kuwait University Anxiety Scale (KUAS) [4] screens for anxiety symptoms and has been validated chiefly among student or non-clinical samples across several Arab countries [5, 6]. A 14-item Perceived Stress Scale (PSS) measuring past-month stress levels and appraisals [7] has been validated in Jordan among teachers and technical workers [8] and in a small sample of Lebanese pregnant women, postpartum women, and students [9]. The 10-item Edinburgh Postnatal Depression Scale (EPDS) [10] has been validated in small samples of postpartum women in the United Arab Emirates (UAE) [11] and Lebanon [12].

While prior studies have validated Arabic translations of the KUAS, PSS, and EPDS in adult samples of men and women, the objective of this study is to validate these scales in a targeted sample of pregnant Arab women living in Qatar, to support prenatal mental health assessment and to facilitate appropriate treatment.

## Methods

### Sample

Arab women over 18 years waiting for prenatal appointments at the Women’s Hospital of Hamad Medical Corporation (Qatar) were recruited. English forms were translated into Qatari Arabic and back-translated by a separate translator to ensure their standard administration. Prior to data collection, cognitive interviews were conducted by trained interviewers among women varied on demographic attributes to ensure local acceptance of topics, wording, and item order. Informed consent and quantitative interviews that covered demographics, resources, agency, and prenatal mental health were administered verbally in language of choice (Arabic or English). The study was approved by the institutional review boards of all collaborating institutions; Hamad Medical Corporation, Emory University, and Qatar University. A fixed interval sampling procedure with replacement was used in that every 7th eligible woman was approached, until satisfying the proposed sample size for completed interviews. Supervisors randomly chose starting points between 1 and 7 each day. The interval was chosen based on pilot testing patient flow. Interviews were conducted in private areas of the clinic. Data collection occurred from January through February of 2017. Of 5896 eligible patients, 840 were invited to participate: 667 completed the interview (response rate: 79.1%); 125 women refused participation, 48 interviews were incomplete, and 2 cases were lost due to ineligibility. Most women who declined participation, did so due to lack of time; incomplete interviews occurred when the woman did not return to complete an interview after her appointment. The prenatal mental health module was estimated to take approximately 10–15 min of the full interview (approximately 55 min).

### Measures

#### Kuwait University anxiety scale

The original KUAS is a 20-item scale that captures cognitive/affective anxiety (9 items), subjective anxiety or nervousness (7 items), and somatic anxiety (4 items) [4]. Example items include “I worry over the future,” “I feel jittery,” and “My heart beats fast.” Original response options were a four point Likert-type intensity scale anchored by *rarely* (=1) and *always* (=4). For this analysis, we recoded response options as *never* (=0), *rarely/sometimes* (=1), *fairly often/always* (=2). Collapsing response options for all measures was an effort to retain ordinality while reducing statistical noise caused by synonymous, quantifiably indistinguishable—and for the EPDS, inconsistent—middle response options [13]. The scale has shown good internal consistency (Cronbach alpha from .70 to .93 test-retest) [4]. In 3064 college students across 10 Arab countries (Egypt, Emirates, Iraq, Jordan, Kuwait, Lebanon, Oman, Palestine, Saudi Arabia, and Syria) [6], the scale had high loadings on all three factors, respectively (> .4, .55–.92, .67–.76). Our study dropped one item, *I am a nervous person,* based on recommendations in the literature to avoid interitem redundancy (with item, *I feel nervous*). for a total of 19 items [14]. Internal reliability was adequate in this sample (α = .819; *n =* 330).

#### Perceived stress scale

The revised PSS is a two-factor 10-item tool that measures one’s appraisal of life events as stressful and was originally validated among a probability U. S. sample with good internal consistency (Chronbach’s alpha = .78)) [15]. Factors align with statements worded negatively (*n* = 6) and positively (*n* = 4). Sample items include: “In the past month, how often have you … felt nervous or stressed?; felt that things were going your way?; and, been upset because of something that happened?” Original responses were Likert-type frequency options anchored by *never* (=1) and *very often* (=5) and recoded as *never* (=0)*, rarely/sometimes* (=1)*, fairy often/very often* (=2) in this study. The PSS-10 has been validated across diverse Arab samples, including perinatal women, with adequate internal consistency (Cronbach alpha = .74–.80); its’ factors have been described as “perceived helplessness” and “perceived self-efficacy” [8, 9]. In our sample, items worded negatively were reversed and the internal reliability of an 8-item scale was adequate (α = .776; *n* = 331).

#### Edinburgh postnatal depression scale

The EPDS is a single factor 10-item measure intended to screen for perinatal depression originally developed among Scottish mothers [10]. Participants respond with varied Likert-type frequency options ranging from *never/not at all/as usual* (=0) to *most of the time*/*quite a lot/quite often/not as usual* (=3) across items such as “In the past week … I have felt sad or miserable; I have looked forward with enjoyment to things; and I have been so unhappy that I have had difficulty sleeping.” Middle response options were collapsed for this study and anchored by *never/not at all/as usual* (=0) and *most of the time/quite a lot/quite often*/*not as usual* (=2). The EPDS has been validated and utilized in diagnostic contexts in the United Arab Emirates (UAE) (Chronbach’s alpha = .84) and Lebanon [11, 12]. Internal reliability was adequate in the sample (α = .753; *n* = 330).

### Procedure

Analysis for the current study used exploratory factor analysis (EFA) and confirmatory factor analysis (CFA), conducted separately for each scale in random split-half samples (*n* = 336; *n* = 331). Conducting EFA/CFA in random split samples is a routine way to explore factor patterns and test the dimensionality and fit of a factor model in two independent samples. EFA assessed scale dimensionality and item factor loadings. CFA tested the factor structure of the final EFA models, and factors were extracted by mean- and variance-adjusted weighted least squares method. Factor loadings were evaluated using standard model fit indices: root mean square error of approximation (RMSEA *close* to 0.060 or lower), comparative fit index (CFI *close* to 0.950 or higher), and Tucker-Lewis index (TLI *close* to 0.950 or higher) after GEOMIN oblique type rotation. Items were removed if they loaded negatively on a factor, loaded < 0.300, or cross-loaded > ǀ0.300ǀ on another factor. MPlus 7.3 [16] and IBM SPSS 24 [17] statistical software were used for all analyses.

## Results

Descriptive characteristics of the participants are shown in Table 1. On average women were 30 years old (SD = 5.08), married in their early 20s to husbands in their late 20s, had three pregnancies, and had two viable births. The sample was predominantly in their 3rd trimester of pregnancy and a majority had attained at least some secondary education or vocational training. Significant differences were observed between Qatari and non-Qatari women. In general, Qatari women were younger at time of interview, married earlier to younger husbands, and had higher numbers of both pregnancies and viable births. Non-Qatari women were more likely to have both secondary/vocational and university/graduate education compared to Qatari women.

**Table 1:** Sample characteristics, pregnant women, Doha, Qatar

	All Women *N* = 667				Qatari *n* = 249				Non-Qatari *n* = 418				Qatari vs. Non-Qatari		
	*n* (%)	*M*	*SD*	Range	*n* (%)	*M*	*SD*	Range	*n* (%)	*M*	*SD*	Range	*t* (df)	*p*	*d*
Age	667 (100)	30.12	*5.08*	19–46	249 (100)	29.41	*5.37*	19–46	418 (100)	30.53	*4.86*	19–45	−2.77 (665)	**	−0.21
Age at marriage^ab^	667 (100)	23.63	*4.20*	13–43	249 (100)	22.71	*4.16*	14–43	418 (100)	24.21	*4.13*	13–39	−4.48 (665)	***	−0.35
Husband’s age at 1st marriage^a^	662 (99)	28.23	*5.14*	18–55	248 (100)	26.40	*5.03*	18–55	414 (99)	29.33	*4.88*	18–51	−7.40 (660)	***	−0.58
Number pregnancies	667 (100)	3.28	*2.09*	1–14	184 (74)	4.47	*2.04*	2–12	303 (73)	3.71	*1.87*	2–14	3.33 (665)	**	0.26
Number live births	529 (79)	2.14	*1.45*	0–7	184 (74)	2.72	*1.58*	1–7	303 (73)	2.09	*0.07*	1–7	5.11 (527)	***	0.45
Number sons	487 (73)	1.17	*0.99*	0–4	184 (74)	1.36	*1.28*	0–4	303 (73)	1.06	*0.88*	0–4	3.35 (485)	**	0.30
Number daughters	487 (73)	1.16	*1.12*	0–6	184 (74)	1.36	*1.24*	0–6	303 (73)	1.05	*1.02*	0–5	3.02 (485)	**	0.27
													−2.77 (665)	**	−0.21
													χ2 (df)	*p*	
Stage of Pregnancy	667 (100)	1.65	*0.59*	0–2	249 (100)	1.67	*0.04*	0–2	418 (100)	1.63	*0.60*	0–2	1.01 (2)	ns	
1st trimester (=0)	41 (6)				15 (6)				26 (6)						
2nd trimester (=1)	153 (23)				52 (21)				101 (24)						
3rd trimester (=2)	473 (71)				182 (73)				291 (70)						
Schooling Attainment, 3 levels	658 (99)	0.76	*0.56*	0–2	244 (98)	0.59	*0.54*	0–2	414 (99)	0.86	*0.56*	0–2	34.91 (2)	***	
None/primary/preparatory (=0)	205 (31)				107 (44)				98 (24)						
Secondary/vocational (=1)	409 (61)				131 (54)				278 (67)						
University/graduate (=2)	44 (7)				6 (3)				38 (9)						

Notes: Ns vary due to refusals, *don’t knows*, and skip patterns. Follow-up chi-squares applying Bonferroni’s correction revealed significant differences between Qatari and non-Qatari women across levels of education such that Qatari women were more likely to have no/primary/preparatory education and non-Qatari women were more likely to have both secondary/vocational and university/graduate education

^a^Refers to age at current / former marriage

^b^No aqid Qaran

*p < 0.05, **p < 0.01, ***p < 0.001

Among sample women (*N* = 667), the average KUAS sum score was 16.06 (range: 0.00–38.00, 38.00 possible; *M =* 0.85; SD = 7.73). On average, PPS-10 mean scores were 1.03 (SD = 0.33) and the average sum score was 10.29 (range: 0.00–19.00; 20.00 possible). Women’s mean EPDS score was 0.54 (SD = 0.36), on average, with sum scores ranging from 0.00 to 17.00 of 20.00 possible (*M* = 5.42; *SD* = 3.61). Descriptive statistics at scale level are presented in Table 2. Item-level descriptive results and inter-item correlations for each scale are available upon request.

**Table 2:** Results of the confirmatory factor analysis for the KUAS, PPS, and EPDS (*N* = 331)

Scale and Factors	*M*	*SD*	Total	1	2
KUAS			1.000	–	–
Cognitive/Affective Anxiety	12.63	±6.53		1.000	–
Somatic Anxiety	2.56	±1.87		0.523***	1.000
PSS	8.09	±3.09			
EPDS	5.18	±3.52			

***p < 0.001

Fit statistics and estimated factor loadings for the final EFA and CFA models are presented in Table 3. EFA and CFA fit statistics for the three scales suggested an adequate fit to the data and estimated factor loadings were positive, similar in magnitude, and were significant. The final CFA model for the KUAS yielded data fit for the 19-item, two factor structure: (a) items 1–15, cognitive and affective anxiety, and (b) items 16–19, somatic anxiety.

**Table 3:** Results of the split-half sample analysis for the KUAS, PSS, and EPDS

Model fit statistics	*KUAS*		*PSS*		*EPDS*	
	*EFA n = 336*	*CFA n = 331*	*EFA n = 336*	*CFA n = 331*	*EFA n = 336*	*CFA n = 331*
CFI	0.964	0.942	0.886	0.968	0.954	0.965
TLI	0.954	0.934	0.840	0.953	0.941	0.955
RMSEA	0.072	0.081	0.082	0.071	0.076	0.058
90% CI	0.063–0.080	0.073–0.089	0.061–0.105	0.048–0.095	0.059–0.093	0.039–0.076

Final CFA models also confirmed 8- and 10-item, single-factor structures for the PSS and EPDS, respectively. During EFA estimations, non-loading PSS item 4 and item 7 were dropped sequentially. Too few perceived efficacy items remained to support a 2-factor model of perceived stress [18]. During CFA, the error variances of PSS items 5 and 8 were allowed to correlate based on modification indices. Internal consistency of factors results and complete factor loadings for the three scales can be found in Additional file 1, respectively.

## Discussion

This cross-sectional study validated scales for depression, anxiety, and perceived stress in a diverse sample of pregnant Arab women in Qatar. The validation of scales for these aspects of mental health in Arab pregnant women is critical to ensure appropriate screening, identification, and treatment to reduce the risk of sequelae in women and their children. Our findings offer a useful comparison to mental-health scale validations in other Arab contexts. For the KUAS, studies among Arab [4, 19, 20] and non-Arab samples [21] suggest 3-factor or 2-factor solutions [14], and our results confirmed a 2-factor solution reflecting cognitive/ affective anxiety and somatic anxiety. For the PSS, research suggests a 2-factor solution, conceptualized as perceived helplessness and perceived self-efficacy [21], and our results suggest that one-factor model offers a concise and theoretically justified solution. For the EPDS, studies among non-Arab populations have generally supported 1- or 2-factor solution [22, 23]. In Arab populations, known validity studies lack information about factor structure and are among postpartum (not pregnant) women resident in Lebanon [12] and UAE [11]. Our study confirms that a one-factor solution capturing overall depressive symptomatology offers a reasonable solution.

In the present study, cognitive interviewing ensured item clarity and cultural appropriateness. Differences in factor structures between our study and prior work may be explained by measures that are insufficiently validated in Arab women (PSS, EPDS), intended to assess depressive symptomology among *postpartum* women (KUAS, EPDS), or originating from a English-language Western context (PSS, EPDS). The saliency of depressive symptoms may differ across postpartum and prenatal women (e.g., different emphasis on somatic items) [24] or across normal and high-risk pregnancies (e.g., different emphasis on anxiety) [25].

Notably, the cross-sectional design precluded an assessment of scale stability over time and a clinic-based sample limits generalizability to pregnant women resident in Qatar. Also, single-country analyses limit generalization to populations in dissimilar contexts. Finally, collapsed response options may miss nuances in some women’s experiences. Despite these caveats, the findings are based on a fixed-interval sample of Qatari and non-Qatari Arab women of diverse socioeconomic levels attending the country’s largest health care provider responsible for caring for the vast majority of births in Qatar, and the response rate was high, at 80%. Scales should be validated in cohorts of pregnant women, to assess stability over time.

## Conclusion

This analysis fills a critical gap in knowledge by rigorously testing scale dimensionality and factor structure of three mental health scales in a diverse sample of pregnant Arab women. Mental health care often is lacking or insufficiently integrated into antenatal care in Arab settings, including Qatar. Most cases of postnatal depression are preceded by antenatal depression [26], thus identification during pregnancy is important. The KUAS, PSS, and EPDS already are known in the health community and their use among pregnant women can be a practical extension for the assessment of mental health. Having well validated Arabic-language scales to capture different dimensions of mental health with due attention to cultural context is critical for the systematic implementation of mental health assessments in prenatal care. In turn, enhanced measurement will lead to wider recognition of mental health issues in pregnancy, their social and biological determinants, and more timely and appropriate prevention and management. Findings also offer a useful comparison to mental-health scale validations in other Arab contexts.

## Supplementary information

**Additional file 1: Table S1.** Internal consistency of factors for KUAS, PSS, and EPDS. **Table S2.** Complete factor loading results based on split half CFA (*n* = 331).

## Data Availability

The datasets used and/or analyzed during the current study are available from the corresponding author on reasonable request.

## Abbreviations

CFA: Confirmatory Factor Analysis
CFI: Comparative Fit Index
CI: Confidence Interval
EFA: Exploratory Factor Analysis
EPDS: Edinburgh Postnatal Depression Scale
KUAS: Kuwait University Anxiety Scale
PSS: Perceived Stress Scale
RMSEA: Root Mean Square Error Of Approximation
TLI: Tucker-Lewis index
UAE: United Arab Emirates

## References

[1] World Health Organization (2019). Mental health: maternal and child mental health.

[2] Yount KM, Smith SM. Gender and postpartum depression in Arab middle eastern women. Women’s Stud Int Forum. 2012;35:187–98.

[3] Fisher JRW, Morrow MM, Nhu-Ngoc MT, Hoang Anh LT (2004). Prevalence, nature, severity and correlates of postpartum depressive symptoms in Vietnam. BJOG Int J Obstet Gynaecol.

[4] Abdel-Khalek AM (2000). The Kuwait university anxiety scale: psychometric properties. Psychol Rep.

[5] Abdel-Khalek AM (2004). Divergent, criterion-related, and discriminant validities for the Kuwait university anxiety scale. Psychol Rep.

[6] Abdel-Khalek AM (2007). Factorial structure of the Kuwait university anxiety scale in a large sample of college students rejoinder. Psychol Rep.

[7] Cohen S, Kamarack T, Mermelstein R (1983). A global measure of perceived stress. J Health Soc Behav.

[8] Almadi T, Cathers I, Hamdan Mansour AM, Chow CM (2012). An Arabic version of the perceived stress scale: translation and validation study. Int J Nurs Stud.

[9] Chaaya M, Osman H, Naassan G, Mahfoud Z (2010). Validation of the Arabic version of the cohen perceived stress scale (PSS-10) among pregnant and postpartum women. BMC Psychiatry..

[10] Cox JL, Holden JM, Sagovsky R (1987). Detection of postnatal depression: development of the 10-item Edinburgh postnatal depression scale. Br J Psychiatry.

[11] Ghubash R, Abou-Selah MT, Daradkeh TK (1997). The validity of the Arabic Edinburgh postnatal depression scale. Soc Psychiatry Psychiatr Epidemiol.

[12] El-Hachem C, Rohayem J, Khalil RB, Richa S, Kesrouani A, Gemayel R (2014). Early identification of women at risk of postpartum depression using the Edinburgh postnatal depression scale (EPDS) in a sample of Lebanese women. BMC Psychiatry.

[13] Knapp TR (1990). Treating ordinal scales as interval scales: an attempt to resolve the controversy. Nurs Res.

[14] Abdel-Khalek AM, Lester D (2003). The Kuwait university anxiety scale: a cross-cultural evaluation in Kuwait and United States. Psychol Rep.

[15] Cohen S, Williamson GM, Spacapam S, Oskamp S (1988). Perceived stress in a probability sample of the United States. The social psychology of health.

[16] Muthén LK, Muthén BO (2012). Mplus user’s guide: statistical analysis with latent variables.

[17] IBM (2015). SPSS statistics for Windows. Version 23.0.

[18] Kline RB. Principles and practice of structural equation modeling. 4th ed. New York: Guilford Press; 2016.

[19] Eid GK, Abdel-Khalek AM. A confirmatory factor analysis of the kuwait university anxiety scale. Soc Behav Pers. 2008;36:399–406.

[20] Abdel-Khalek AM, Al-Damaty A-GA (2003). The Kuwait university anxiety scale: results for 9,031 saudi students. Psychol Rep.

[21] Crandall A, Abdul-Rahim HF, Yount KM (2015). Validation of the general self-efficacy scale in adolescent qatari women. East Mediterr Health J.

[22] De Bruin GP, Swartz L, Tomlinson M, Cooper PJ, Molteno C (2004). The factor structure of the Edinburgh postnatal depression scale in a south African peri-urban settlement. S Afr J Psychol.

[23] Montazeri A, Torkan B, Omidvari S (2007). The Edinburgh postnatal depression scale (EPDS): translation and validation study of the Iranian version. BMC psychiatry.

[24] Matthey S, Henshaw C, Elliott S, Barnett B (2006). Variability in use of cut-off scores and formats on the Edinburgh postnatal depression scale–implications for clinical and research practice. Arch Women’s Ment Health.

[25] Adouard F, Glangeaud-Freudenthal NM, Golse B (2005). Validation of the Edinburgh postnatal depression scale (EPDS) in a sample of women with high-risk pregnancies in France. Arch Women’s Ment Health.

[26] Heron J, O’Connor TG, Evans J, Golding J, Glover V, ALSPAC study team. The course of anxiety and depression through pregnancy and the postpartum in a community sample. J Affect Disord. 2004;80:65–73. 10.1016/j.jad.2003.08.004 15094259

