# Supplementary material for: Validation of three mental health scales among pregnant women in Qatar
Source: Reprod Health. 2019 Oct 16;16:149. doi: 10.1186/s12978-019-0806-6 (PMC6796339; doi:10.1186/s12978-019-0806-6)
Supplement: Supplementary file 1 — Additional file 1: Table S1. Internal consistency of factors for KUAS, PSS, and EPDS. Table S2. Complete factor loading results based on split half CFA (n = 331). [file 12978_2019_806_MOESM1_ESM.docx]

Article Title: Validation of three mental health scales among pregnant women in Qatar

Journal Title: Reproductive Health

Supplementary Tables

**Table S1** Internal consistency of factors for KUAS, PSS, and EPDS

| Factor Name Chronbach’s α | Item | α if item deleted | Corrected item-total correlations |
| --- | --- | --- | --- |
|  | KUAS (α = .819; *n* = 330) | |  |
| Cognitive/ | (1) I worry over the future. | 0.901 | 0.486 |
| Affective | (2) I am not at ease. | 0.900 | 0.529 |
| Anxiety | (3) I feel unstable. | 0.898 | 0.589 |
| α = .904 | (4) I am restless. | 0.901 | 0.490 |
|  | (5) I feel frightened. | 0.899 | 0.558 |
|  | (6) I feel worried. | 0.899 | 0.554 |
|  | (7) I think of things that worry me. | 0.896 | 0.624 |
|  | (8) I expect bad things to happen. | 0.897 | 0.601 |
|  | (9) I feel anxious. | 0.895 | 0.674 |
|  | (10) I feel jittery. | 0.897 | 0.614 |
|  | (11) My nerves are strained. | 0.894 | 0.670 |
|  | (12) I feel nervous. | 0.894 | 0.668 |
|  | (13) I am tense. | 0.893 | 0.708 |
|  | (14) My muscles feel tense. | 0.899 | 0.543 |
| Somatic | (15) I am afraid of losing self-control. | 0.899 | 0.548 |
| Anxiety | (16) My heart beats fast. | 0.643 | 0.483 |
| α = .703 | (17) I suffer from short breath. | 0.611 | 0.532 |
|  | (18) My heart beats are irregular. | 0.629 | 0.510 |
|  | (19) I get dizzy. | 0.674 | 0.433 |
|  | PSS (α = .776; *n* = 331) | |  |
|  | *In the past month, how often have you…* |  |  |
|  | (1) been upset because of something that happened? | 0.744 | 0.521 |
|  | (2) felt unable to control the important things in life? | 0.735 | 0.568 |
|  | (3) felt nervous and “stressed”? | 0.749 | 0.494 |
|  | (5) felt that things were going your way? | 0.759 | 0.426 |
|  | (6) found that you could not cope with all the things that you had to do? | 0.771 | 0.351 |
|  | (8) felt that you were on top of things? | 0.760 | 0.426 |
|  | (9) been angered because of things that were outside of your control? | 0.750 | 0.490 |
|  | (10) felt difficulties were piling up so high that you could not overcome them? | 0.741 | 0.533 |
|  | EPDS (α = .753; *n* = 330) | |  |
|  | *In the past week...* | |  |
|  | (1) I have been able to laugh and see the funny side of things. | 0.749 | 0.275 |
|  | (2) I have looked forward with enjoyment to things. | 0.745 | 0.322 |
|  | (3) I have blamed myself unnecessarily when things went wrong. | 0.737 | 0.398 |
|  | (4) I have been anxious or worried for no good reason. | 0.734 | 0.411 |
|  | (5) I have felt scared or panicky for no good reason. | 0.722 | 0.491 |
|  | (6) Things have been getting on top of me. | 0.741 | 0.369 |
|  | (7) I have been so unhappy that I have had difficulty sleeping. | 0.716 | 0.519 |
|  | (8) I have felt sad or miserable. | 0.702 | 0.600 |
|  | (9) I have been so unhappy that I have been crying. | 0.710 | 0.562 |
|  | (10) The thought of harming myself has occurred to me. | 0.759 | 0.137 |

Note. One case was dropped from scale reliability analysis due to missing data on item 3 (EPDS) and items 8 and 14 (KUAS).

**Table S2** Complete factor loading results based on split half CFA (*n* = 331)

| KUAS | | |
| --- | --- | --- |
|  | Factor loading | |
| (#) Item | Factor 1 | Factor 2 |
| (1) I worry over the future. | **.555** |  |
| (2) I am not at ease. | **.661** |  |
| (3) I feel unstable. | **.683** |  |
| (4) I am restless. | **.583** |  |
| (5) I feel frightened. | **.658** |  |
| (6) I feel worried. | **.655** |  |
| (7) I think of things that worry me. | **.739** |  |
| (8) I expect bad things to happen. | **.709** |  |
| (9) I feel anxious. | **.810** |  |
| (10) I feel jittery. | **.777** |  |
| (11) My nerves are strained. | **.832** |  |
| (12) I feel nervous. | **.849** |  |
| (13) I am tense. | **.831** |  |
| (14) My muscles feel tense. | **.680** |  |
| (15) I am afraid of losing self-control. | **.661** |  |
| (16) My heart beats fast. |  | **.788** |
| (17) I suffer from short breath. |  | **.747** |
| (18) My heart beats are irregular. |  | **.687** |
| (19) I get dizzy. |  | **.584** |
| PSS | | |
| (#) Item | Factor 1 |  |
| *In the past month, how often have you…* |  |  |
| (1) been upset because of something that happened? | **.695** |  |
| (2) felt unable to control the important things in life? | **.739** |  |
| (3) felt nervous and “stressed”? | **.669** |  |
| (5) found that you could not cope with all the things that you had to do? | **.489** |  |
| (6) felt that you were on top of things? | **.468** |  |
| (8) felt difficulties were piling up so high that you could not overcome them? | **.491** |  |
| (9) been upset because of something that happened? | **.682** |  |
| (10) felt unable to control the important things in life? | **.713** |  |
| EPDS | | |
| (#) Item | Factor 1 |  |
| *In the past week,* |  |  |
| (1) I have been able to laugh and see the funny side of things. | **.471** |  |
| (2) I have looked forward with enjoyment to things. | **.499** |  |
| (3) I have blamed myself unnecessarily when things went wrong. | **.508** |  |
| (4) I have been anxious or worried for no good reason. | **.554** |  |
| (5) I have felt scared or panicky for no good reason. | **.648** |  |
| (6) Things have been getting on top of me. | **.462** |  |
| (7) I have been so unhappy that I have had difficulty sleeping. | **.663** |  |
| (8) I have felt sad or miserable. | **.817** |  |
| (9) I have been so unhappy that I have been crying. | **.786** |  |
| (10) The thought of harming myself has occurred to me. | **.651** |  |

Note. Factors were extracted by mean- and variance-adjusted weighted least

squares method with geomin (oblique) rotation.
